# Exploring Factors Associated With Mobile Phone Behaviors and Attitudes Toward Technology Among Adults With Alcohol Use Disorder and Implications for mHealth Interventions: Exploratory Study

**DOI:** 10.2196/32768

**Published:** 2022-08-15

**Authors:** Marie Aline Sillice, Michael Stein, Cynthia L Battle, Lidia Z Meshesha, Clifford Lindsay, Emmanuel Agu, Ana M Abrantes

**Affiliations:** 1 City University of New York School of Public Health & Health Policies Center for Systems and Community Design New York, NY United States; 2 Department of Health Law, Policy, and Management Boston University Boston, MA United States; 3 Department of Psychiatry and Human Behavior Warren Alpert School of Medicine of Brown University Providence, RI United States; 4 Department of Psychology University of Central Florida Orlando, FL United States; 5 Department of Radiology University of Massachusetts Medical School Worcester, MA United States; 6 Computer Science Department Worcester Polytechnic Institute Worcester, MA United States

**Keywords:** mobile phone use patterns, substance use, alcohol, technological attitude, alcohol use disorder, demographic differences, anxiety, depression, mobile phone, patient attitude

## Abstract

**Background:**

Alcohol use disorder (AUD) is associated with severe chronic medical conditions and premature mortality. Expanding the reach or access to effective evidence-based treatments to help persons with AUD is a public health objective. Mobile phone or smartphone technology has the potential to increase the dissemination of clinical and behavioral interventions (mobile health interventions) that increase the initiation and maintenance of sobriety among individuals with AUD. Studies about how this group uses their mobile phone and their attitudes toward technology may have meaningful implications for participant engagement with these interventions.

**Objective:**

This exploratory study examined the potential relationships among demographic characteristics (race, gender, age, marital status, and income), substance use characteristics (frequency of alcohol and cannabis use), and clinical variables (anxiety and depression symptoms) with indicators of mobile phone use behaviors and attitudes toward technology.

**Methods:**

A sample of 71 adults with AUD (mean age 42.9, SD 10.9 years) engaged in an alcohol partial hospitalization program completed 4 subscales from the Media Technology Usage and Attitudes assessment: *Smartphone Usage* measures various mobile phone behaviors and activities, *Positive Attitudes* and *Negative Attitudes* measure attitudes toward technology, and the *Technological Anxiety/Dependence* measure assesses level of anxiety when individuals are separated from their phone and dependence on this device. Participants also provided demographic information and completed the Epidemiologic Studies Depression Scale (CES-D) and the Generalized Anxiety Disorder (GAD-7) scale. Lastly, participants reported their frequency of alcohol use over the past 3 months using the Drug Use Frequency Scale.

**Results:**

Results for the demographic factors showed a significant main effect for age, *Smartphone Usage* (*P*=.003; *η*_p_^2^=0.14), and *Positive Attitudes* (*P*=.01; *η*_p_^2^=0.07). Marital status (*P*=.03; *η*_p_^2^=0.13) and income (*P*=.03; *η*_p_^2^=0.14) were associated only with the Technological Anxiety and Dependence subscale. Moreover, a significant trend was found for alcohol use and the *Technological Anxiety/Dependence* subscale (*P*=.06; *R*^2^=0.02). Lastly, CES-D scores (*P*=.03; *R*^2^=0.08) and GAD symptoms (*P*=.004; *R*^2^=0.13) were significant predictors only of the *Technological Anxiety/Dependence* subscale.

**Conclusions:**

Findings indicate differences in mobile phone use patterns and attitudes toward technology across demographic, substance use, and clinical measures among patients with AUD. These results may help inform the development of future mHealth interventions among this population.

## Introduction

### Background

Many chronic health problems are associated with alcohol use disorder (AUD), including stroke, high blood pressure, heart disease, cancer of the esophagus, liver, and colon [1-3]. AUD is also related to a plethora of psychological and behavioral problems [3]. According to the 2019 National Survey on Drug Use and Health, prevalence rates of AUD among US adults show that 14.1 million have this disorder [4]. Approximately 88,000 people die annually from alcohol-related diseases [4,5], making it a significant public health concern. Accordingly, an objective of alcohol treatments is to help patients abstain from alcohol use.

Long-term abstinence has been shown to improve various complications of alcohol-related diseases [6]. Abstinence maintenance is intricately linked with the successful completion of the initial days following alcohol cessation, when individuals tend to experience elevated anxiety, depression, and cravings for alcohol and are thus at high risk for a relapse occurrence [7,8]. Patients at this stage tend to report low self-efficacy to effectively manage daily triggers for alcohol consumption [9,10]. Consequently, fundamental strategies to facilitate the acquisition of sobriety and long-term maintenance requires real-time interventions that provide individuals with ongoing support and the necessary skills to manage relapse risk factors [9,10]. Mobile phone technologies are among the recommended platforms to augment public health impact [11,12] and can be harnessed to increase reach and dissemination of multifaceted approaches designed to effectively address both cognitions and behaviors associated with AUD.

The wide availability of mobile phone or smartphone ownership (97%) and frequent app usage (80% in the past 30 days) among US adults [13,14] provides an opportunity for researchers to reach this population at scale. Traditional face-to-face substance use intervention programs are inherently limited in their ability to assess and treat real-time risks that can occur in the individual's day-to-day environment [11]. Moreover, low engagement and high attrition are common among traditional substance use treatment interventions with this population, particularly among patients in early recovery [11,15,16]. Smartphone-delivered interventions have shown promising results in increasing engagement and improving outcomes with mental health and behavioral health treatments, although most mHealth studies lack any theoretical framework [11,17,18]. For example, a recent review shows efficacy of mHealth alcohol use interventions, but results remain mixed overall [11].

According to Golbert et al [11], strengthening the rigor of this emerging research requires applying theoretically informed approaches and the use of randomized controlled trials (RCTs) to adequately assess the effect of these new interventions [11]. To our knowledge, there are 2 theory-informed, smartphone app delivered interventions for adults with AUD being conducted [19,20]. While these studies could help determine the efficacy of these technology-based approaches for improving alcohol use outcomes, essential to their success is an understanding of how individuals with AUD use their mobile phone in their daily lives and their attitudes toward technology, which may have an effect on participants' engagement with these interventions.

Studies conducted with general populations have demonstrated variability in mobile use patterns across demographic subgroups (eg, men vs women, White vs non-White, and married vs single) [21,22]. Correspondingly, these findings have been used to inform the development of mHealth approaches addressing barriers and facilitators for behaviors that are more likely to appeal to particular groups [21,22]. Similar assessment studies with individuals with AUD may provide guidance for the development of mHealth intervention approaches in different subgroups of this population, such as those with higher levels of comorbid affective symptoms (eg, anxiety and depression). Because mHealth interventions with individuals with AUD is a developing research area, examining predictors of smartphone use and attitudes toward technology is an important step toward advancing this work.

### Objectives

This study explores mobile use behaviors and attitudes toward technology among adults with AUD receiving outpatient treatment. In addition to demographic characteristics (eg, age, gender, and marital status), mental health factors (eg, anxiety and depression symptoms), which are highly relevant to this population [23,24], were also examined as potential correlates of mobile phone behaviors and attitudes toward technology. Moreover, a potential effect of the level of alcohol use on mobile phone behaviors and attitudes toward technology was explored.

## Methods

### Participant Recruitment and Study Design

Participants were recruited from an alcohol and drug partial hospitalization program at a private hospital in the Northeastern United States. This program provides an abstinence-based and cognitive-behavioral treatment. Patients attend 3-4 groups per day (eg, relapse prevention, drink, drug refusal skills, goal-setting, etc), daily individual counseling with a mental health worker, and medication management with an attending psychiatrist. Adult patients were approached by research staff to determine their interest in participating in a study designed to develop or test a 12-week smartphone app for increasing physical activity engagement among adults in early recovery from alcohol. Recruitment occurred in 2 phases: as part of an open pilot and then subsequently as part of a RCT. Data collected as part of the baseline assessment from each of these phases were examined in this paper.

### Ethics Approval

The study was approved by the Institutional Review Board at Butler Hospital (IRB# 1604-003).

### Measures

Demographic information was collected for race, age, ethnicity, gender, marital status, income, and education.

#### Media and Technology Usage and Attitudes (MTUA) Scale

The MTUA is a 50-item scale with 15 subscales. In this study, we administered 4 subscales: *Smartphone Usage*, *Positive Attitudes Toward Technology*, *Negative Attitudes Toward Technology*, and *Technological Anxiety/Dependence*. Each subscale has been shown to have strong validity and reliability [25]. The *Smartphone Usage* subscale consists of 7 items assessing the frequency, on a 10-point frequency, ranging from 1=never to 10=all of the time, of engaging in various smartphone activities (eg, texting, emailing, taking pictures). The other 3 subscales are measured based on a Likert-type scale, ranging from 1=strongly agree to 5=strongly disagree [25] and assess different attitudes toward technology: (1) *Positive Attitudes* subscale (an item is “I feel I get more accomplished because of technology”), (2) *Negative Attitudes* subscale (an item is “new technology makes life more complicated”), and (3) *Technological Anxiety/Dependence* subscale measures anxiety that resulted from individuals being away from their phone (an item is “I get anxious when I don’t have my phone with me”).

#### The Drug Use Frequency Scale

The Drug Use Frequency Scale is a self-report instrument consisting of 10 items that measure the frequency of use for different substances over the past 3 months [26]. Participants reported their frequency of alcohol use (and use of other substances) using a 7-point Likert-type scale ranging from 0=not all to 7=every day. A score of ≥5 indicates a high frequency of substance use [26].

#### Generalized Anxiety Disorder 7-item (GAD-7)

The GAD-7 scale consists of items that reflect the diagnostic symptom criteria for this disorder (eg, “feeling anxious, nervous, and on edge”) based on the Diagnostic and Statistical Manual of Mental Disorders, Fourth Edition [27]. Participants are asked to indicate how often in the last 2 weeks they were bothered by the different symptoms. Response options are 0=not at all to 3=nearly every day. Items 1-7 are summed to provide a total score [27].

#### The Center for Epidemiologic Studies Depression (CES-D) Scale

CES-D is a 20-item self-report measure that assesses the presence of depression symptoms experienced over the past week [28]. Each item is measured on a 4-point Likert-type scale that indicates the frequency of depression symptoms ranging from 0=rarely or none of the time to 3=most or all of the times. A sum of scores is calculated for this measure [28].

### Statistical Methods

#### Overview

Frequencies for the following demographic characteristics were examined in the combined data set from the 2 trials (open pilot and RCT): race, marital status, age, gender, ethnicity, education, employment, and income. There were missing data for variables (eg, employment, education, and income) not collected in the open pilot study.

#### Sample Characteristics and Development of Subgroups

Sample size constraints for the variables race and marital status allowed for comparison between being White and non-White and married/living with a partner vs single/divorced/widowed. For age, a median split approach was used to create 2 age groups: ≥43 years and ≤44 years. Less than 1% of the sample reported an ethnic identity, and hence group comparisons were not feasible. Employment status was coded into 2 groups: employed vs unemployed/retired/disabled. For education, the groups were high school/some college versus college degree/advanced degrees. Annual income was reported by 39 participants and was coded into 2 categories: ≤US $75,000 and ≥US $75,000.

#### Preliminary Analyses

Chi-square tests were used to explore potential proportion difference among race, gender, age (independent variables), and marital status, education, employment, and annual income (dependent variables). A series of ANOVA models evaluated mean differences for the abovementioned 7 demographics and the 3 dependent variables, GAD-7, CES-D, and alcohol use.

#### Primary Analyses

ANOVA evaluated demographic differences for each of the 4 MTUA subscales. Separate linear regression analyses were used to explore a potential association between anxiety (GAD-7) and depression (CES-D), alcohol use (independent variables), and each of the MTUA subscales (dependent variables). In addition to statistical significance, partial eta-squared (*η*_p_^2^), *R*, or *R*^2^ *β* values were used, as appropriate, to demonstrate the level of association between the independent variables and dependent measures [29,30].

## Results

### Sample Description

The majority of participants identified as White (56/70, 80%), others identified as non-White (14/70, 20%), and over half of the participants were men (36/62, 58%). Nearly half of the sample was aged ≤43 years (30/61, 49%), while 51% (31/61) of participants were aged ≥44 years. Participant age range was 20-64 (mean 42.89, SD 10.9) years. Fifteen participants were married/living with a partner (15/40, 38%), and 25 (63%) were single/divorced/widowed. For education, 43% (17/40) of participants were in the high school/some college category, and 57% (23/40) were in the college degree/advanced degrees group. Moreover, 56% (27/48) of the sample was employed, and 12% (25/48) were unemployed/retired/disabled. Furthermore, 67% (24/39) of participants reported an annual income of ≤US $75,000, while 39% reported an annual income of ≥US $75,000. The distribution of alcohol use variables showed that 37% (26/71) of the sample reported consuming alcohol for “5-6 days a week” over the past 3 months. A higher number of participants (33/71, 47%) reported consuming alcohol “every day” over the past 3 months.

There were no statistically significant differences for race, gender, age and marital status, education, employment, and annual income (Table 1).

Moreover, there were no statistically significant relationships between the demographics and the GAD-7, CES-D, and alcohol use variables (see Table 2).

**Table 1:** Statistics for the seven demographic subgroups.

	Race				Gender				Age		
	Chi-square (*df*)	Participants, n	*P* value	Chi-square (*df*)		Participants, n	*P* value	Chi-square (*df*)		Participants, n	*P* value
Marial status	1.307 (1)	40	.74	0.365 (1)		39	.74	0.062 (1)		38	.54
Education	1.687 (1)	40	.37	0.843 (1)		39	.52	0.001 (1)		38	.62
Employment	0.036 (1)	40	.62	0.530 (1)		33	.47	0.516 (1)		38	.75
Annual income	0.399 (1)	39	.66	0.542 (1)		38	.51	0.016 (1)		37	.90

**Table 2:** Inferential statistics on the associations among demographics, generalized anxiety, depression, and alcohol use.

	GAD-7			CES-D			Alcohol use	
	*F* test (*df*)	*P* value	*F* test (*df*)		*P* value	*F* test (*df*)		*P* value
Race	0.480 (1,65)	.49	0.741 (1,61)		.74	0.003 (1,68)		.96
Gender	0.592 (1,56)	.45	0.417 (1,53)		.52	3.705 (1,60)		.06
Age	0.269 (1,55)	.78	0.796 (1,52)		.38	0.269 (1,59)		.61
Marital status	1.188 (1,36)	.28	0.617 (1,33)		.44	0.0001 (1,38)		>.99
Education	0.226 (1,36)	.64	2.075 (1,33)		.16	0.780 (1,38)		.38
Employment	1.969 (1,34)	.51	1.957 (1,34)		.17	1.696 (1,36)		.20
Annual income	0.447 (1,37)	.51	1.527 (1,32)		.23	0.510 (1,37)		.48

### MTUA Subscales and Demographic Characteristics

#### Smartphone Usage Subscale

Smartphone usage scores were significantly different between the 2 age groups (*F*_1,69_=10.87; *P*=.002; *η*_p_^2^=0.14). Participants aged ≤43 years had a higher mean score on this measure (mean 29.46, SD 5.07) than those aged ≥44 years (mean 24.35, SD 7.67). There were no significant relationships for the subscale and the other demographic variables. Detailed information can be found in Table 3.

**Table 3:** Inferential statistics for the demographics, Media Technology Usage and Attitudes subscales, and clinical characteristics.

			*Smartphone Usage*				*Positive Attitude*				*Negative Attitude*				*Technological Anxiety/Dependence*		
			*F* test (*df*)		*P* value		*F* test (*df*)		*P* value		*F* test (*df*)		*P* value		*F* test (*df*)		*P* value
**Demographics**																	
	Race	0.911 (1,68)		.34		0.071 (1,68)		.79		0.190 (1,68)		.66		0.548 (1,67)		.46	
	Gender	2.353 (1,60)		.13		0.847 (1,60)		.36		0.058 (1,60)		.81		0.106 (1,60)		.75	
	Age	10.87 (1,69)		.002		4.819 (1,69)		.03		0.493 (1,68)		.49		0.788 (1,69)		.79	
	Marital status	0.374 (1,38)		.55		2.629 (1,38)		.11		0.352 (1,37)		.56		5.468 (1,38)		.03	
	Education	0.065 (1,38)		.80		3.368 (1,38)		.07		2.586 (1,37)		.12		1.084 (1,38)		.30	
	Employment	0.153 (1,46)		.70		6.196 (1,46)		.02		0.164 (1,45)		.69		1.257 (1,46)		.27	
	Annual income	0.403 (1,37)		.53		0.585 (1,37)		.45		0.583 (1,36)		.45		6.196 (1,37)		.02	
**Clinical characteristics**																	
	Generalized Anxiety Disorder	2.194 (1,65)		.14		0.868 (1,65)		.36		0.331 (1.64)		.57		5.135 (1,65)		.03	
	Epidemiologic Studies Depression Scale	3.554 (1,62)		.06		2.194 (1,65)		.14		0.113 (1,62)		.74		9.024 (1,62)		.004	
	Alcohol use	0.525 (1,69)		.47		0.374 (1,69)		.54		0.046 (1,68)		.83		3.640 (1,69)		.06	

#### Positive Attitudes Subscale

Positive attitudes toward technology were significantly different between the 2 age groups, (*F*_1,69_=4.819; *P*=.03; *η*_p_^2^=0.07). Specifically, younger participants, aged ≤43 years, had a greater positive attitude toward technology (mean 22.75, SD 3.90) than those in the older age group, aged ≥44 years (mean 20.94, SD 2.63). As shown in Table 3, there were no significant relationships for this subscale and the other demographic variables.

#### Technological Anxiety/Dependence Subscale

Marital status was associated with *Technological Anxiety/Dependence* (*F*_1,38_=5.468; *P*=.03; *η*_p_^2^=0.13). Participants who were married reported less anxiety when separated from their phone and less dependence on their device (mean 8.26, SD 3.43) compared to the single, divorced, widow group (mean 10.48, SD 2.54). In addition, differences in scores on the *Technological Anxiety/Dependence* subscale were also observed between the income groups (*F*_1,37_=6.196; *P=.*02; *η*_p_^2^=0.14). Individuals with an annual income of ≤US $75,000 had greater technological anxiety or dependence on their phone (mean 10.63, SD 2.28) versus those with an annual income of ≥US $75,000 (mean 8.27, SD 3.65). There were no significant relationships between the 2 subscales and the other demographic variables (see Table 3).

### MTUA Subscales and Anxiety and Depression Symptoms

Anxiety was a significant predictor of *Technological Anxiety/Dependence* scores (*F*_1,65_=5.135; *P*=.03). The correlation coefficient (*R*=0.27) shows a positive linear relationship between the 2 variables. Anxiety symptoms accounted for 8% (*R^2^*=0.08) of variance in *Technological Anxiety/Dependence* scores. A significant *β* coefficient of .22 (*t*=2.266; *P*=.03), suggesting a one-unit increase of 0.22 in reported anxiety and dependency on technology for every 1-point increase in anxiety, as measured by the GAD-7 scale. Significant findings were not found between anxiety and *Smartphone Usage* (*F*_1,65_=2.194; *P*=.14), *Positive Attitude* (*F*_1,65_=0.868; *P*=.36), and *Negative Attitude* (*F*_1,64_=0.331; *P*=.57).

Depressive symptoms were also a significant predictor of *Technological Anxiety/Dependence* subscale scores (*F*_1,62_=9.024; *P*=.004). The correlation coefficient (*R*=0.36) shows a positive and linear relationship between the 2 variables. Depression symptoms account for 13% (*R^2^*=0.13) of variance in the *Technological Anxiety/Dependence* measure. A significant *β* coefficient of .36 (*t*=3.004; *P*=.004) was noted, indicating for one-unit increase in depression, there is a .36 increase in technological anxiety/dependence. A near significant trend was noted between depression symptoms and *Positive Attitude* (*F*_1,62_=3.554; *P*=.06). Results for the other subscales were as follows: *Smartphone Usage* (*F*_1,62_=1.316; *P*=.26) and *Negative Attitude* (*F*_1,61_=0.113; *P*=.74).

### MTUA Subscales and Alcohol Use

Frequency of alcohol use in the past 3 months and reported anxiety when being away from one’s mobile phone or being dependent on this device showed a near significant trend (*F*_1,69_=3.640; *P*=.06; *R^2^*=0.02). Results for the other subscales were as follows: *Smartphone Usage* (*F*_1,69_=0.525, *P*=.47), *Positive Attitudes* (*F*_1,69_=0.612, *P*=.54), and *Negative Attitudes* (*F*_1,68_=0.046, *P*=.83).

## Discussion

### Principal Findings

This study provides an examination of mobile phone use behavioral patterns and attitudes toward technology among a sample of adults with alcohol use disorder (AUD) in early recovery. Demographics, anxiety and depressive symptoms, and alcohol use were associated with smartphone usage and attitudes toward technology. These results may provide insights into the development of mobile phone delivered intervention (mHealth) approaches for individuals with AUD.

Relative to older patients with AUD, those aged ≤43 years reported higher rates of smartphone usage and were more likely to have positive attitudes about media use. Specifically, younger patients had greater reliance on their mobile phone to complete various tasks, such as using apps, searching for directions, and browsing the web and reported a more positive view of these activities. These results are consistent with previous studies demonstrating a strong association between being a younger age and greater reliance on this device to complete many daily tasks—aided by easy access to the internet—compared to older adults [21,22]. High usage of mobile apps has been shown to be associated with perceived importance in facilitating the accomplishment of targeted goals using these platforms [21,22].

Therefore, this younger subgroup of patients may be very receptive to using a smartphone app to help during early recovery, and mHealth strategies consistent with how this group uses their phone are likely to be more acceptable and engaging. For example, a mobile phone app with a “resource” feature on AUD may provide a menu of information on the psychophysiological impact of this disorder, effective treatments, including strategies for managing risks for relapse, such as environmental triggers, depression, anxiety, and cravings [1,2,4]. Given existing barriers to treatment and the impact of chronic alcohol use on long-term memory and cognitive functioning [6], ready access to this information in moments of greatest need (eg, high-risk situations) may be critical toward improving alcohol treatment outcomes.

Our findings also demonstrated that single/divorced/widowed participants indicated greater anxiety without their phone or feeling more dependent on this device than those who were married/living with a partner. A previous study assessing mobile phone use behaviors among a nonclinical population has shown overall similar results [21]. It is possible that individuals with AUD who do not live with a partner are more likely to rely on their phone to remain connected with family members or friends. Therefore, when developing technology-supported approaches for individuals with AUD not living with partners, app features that allow participants to easily connect with others may be desirable. For example, apps that contain message boards that allow communication between users or being able to use certain *keywords* (eg, “struggling”) to immediately connect with a clinician to receive additional support to address emerging barriers or experiences could be an attractive app feature in this subgroup individuals.

Moreover, participants with an annual income of ≤US $75,000 also showed higher anxiety without their phone or were more dependent on their device. A Pew Research Center report on mobile phone usage and annual income conducted between 2013 and 2021 showed individuals of this income bracket as being more smartphone-dependent than their higher-income counterparts [13]. While it is not clear what contributes to the difference in dependency on smartphones between income groups, this report found individuals of this income level are more likely to be “smartphone only internet users” and less likely to own other devices (eg, a computer or iPad) [13]. It is possible that lower financial resources indicate greater increased reliance to on this device to complete many and different tasks. Moreover, AUD is more prevalent among socioeconomically disadvantaged groups than those of a higher income level [31,32]. This intersection has been associated with a higher prevalence of many chronic conditions, such as liver disease, type 2 diabetes, hypertension, and some cancers, compared to the general population [2,33,34]. The current evidence shows that low-income adults with AUD are significantly dependent on their mobile phone and thus suggests the potential acceptability of mHealth programs among this subgroup. Accordingly, researchers have the opportunity to deliver both clinical and behavioral health intervention approaches that address cognitions and behaviors salient in increasing sobriety and thereby decreasing associated health risks among this group. For example, engagement in physical activity or yoga has shown to be beneficial as an adjunctive tool in treatments for AUD [35-37]; however, these interventions are small and are typically delivered in person and over many months. Accordingly, mHealth approaches have the potential to reach a large section of this group and promote sobriety on a large scale.

Lastly, the study findings showed that anxiety and depression symptoms were significant predictors of *Technological Anxiety/Dependence* scores, although they accounted for minimal variance (*R*^2^=0.08 and 0.13, respectively). Nevertheless, anxiety and depression symptoms are highly prevalent among individuals with AUD, and mobile phone app programs that would allow participants to track their symptoms may provide insights into trends associated, such as the relationship between anxiety or depression symptoms and alcohol cravings. Additionally, an app feature that could enable participants to share this type of information with their provider could help inform treatment decisions. However, more research is necessary to determine whether media anxiety and phone dependency may, in fact, be contributing toward an increase in anxiety and depression symptoms in this population.

### Limitations and Future Work

An important limitation of this study is the lack of heterogeneity with respect to participant ethnicity, preventing an assessment of a potential relationship between a particular ethnic group and mobile phone or media constructs. For example, Latinx populations have increased incidence rates of AUD that may be linked to minority stress and socioeconomic status [31, 32]. Assessment of mobile use behaviors among Latinx adults with AUD is an important research area to determine the potential receptiveness of mHealth substance abuse treatment approaches in this group. Accordingly, more research is needed to extend the current understanding of the nature or frequency of mobile phone usage and views of this technology across different demographic subgroups with AUD. In addition, participants enrolled in this study were interested in using a smartphone app to help them increase their physical activity in early recovery. It is possible that the results of this study may not generalize to the broader AUD patient population. Moreover, future studies with a larger sample size with AUD is needed to assess these relationships. Despite these limitations, the findings may help inform future mHealth approaches that can be used to augment addiction treatment in individuals with AUD. Aligned with the goals of precision medicine, mHealth approaches that are tailored to specific individuals needs and characteristics may be more effective in improving overall treatment outcomes.

### Conclusions

Notwithstanding these limitations, the study findings provide insight into the relationship between age, marital status, income, depression, and anxiety on empirical constructs for mobile phone use behaviors in adults with AUD. Moreover, the study results provide knowledge into mHealth approaches that are likely to appeal to the needs of different demographic adult subgroups with AUD. Our findings accentuate the need to fully understand individuals’ mobile phone use and attitudes toward technology to evaluate their potential influence on the level of engagement with mHealth interventions in different adult groups with AUD.

## Abbreviations

AUD: alcohol use disorder
CES-D: Epidemiologic Studies Depression Scale
GAD-7: Generalized Anxiety Disorder 7-item
mHealth: mobile health
MTUA: Media Technology Usage and Attitudes
RCT: randomized controlled trial
